# Screening for Developmental Disorders in 3- and 4-Year-Old Italian Children: A Preliminary Study

**DOI:** 10.3389/fped.2017.00181

**Published:** 2017-08-29

**Authors:** Elena Catino, Michela Di Trani, Federica Giovannone, Filippo Manti, Letizia Nunziata, Francesca Piccari, Virginia Sirchia, Lucia Vannucci, Carla Sogos

**Affiliations:** ^1^Department of Child Neurology and Psychiatry, Sapienza Università di Roma, Rome, Italy; ^2^Department of Dynamic and Clinical Psychology, Sapienza Università di Roma, Rome, Italy

**Keywords:** children, screening, neurodevelopmental disorders, mental health, epidemiology

## Abstract

**Background:**

The “Osserviamo” project, coordinated by the Municipality of Rome and the Department of Pediatrics and Child Neuropsychiatry of Sapienza University, aimed to validate an Italian version of the Ages and Stages Questionnaire-3 and to collect, for the first time in Italy, data on developmental disorders in a sample of 4,000 children aged 3 and 4 years. The present paper presents the preliminary results of the “Osserviamo” project.

**Methods:**

600 parents of children between 39 and 50 months of age (divided in two age stages: 42 and 48 months) were contacted from 15 kindergarden schools.

**Results:**

23.35% of the whole sample scored in the risk range of at least one developmental area of the Ages and Stages Questionnaire-3rd Edition (ASQ-3) and 7.78% scored in the clinical range. Specifically, 23.97% of the children in the 42-month age stage scored in the risk range and 5.79% scored in the clinical range. Males scored lower than females in the fine motor skills and personal–social development domains. Moreover, 22.79% of the children in the 48-month age stage scored in the risk range, while 9.55% scored in the clinical range. Males scored lower than females in fine motor skills.

**Conclusion:**

Italian validation of the ASQ-3 and recruitment of all 4,000 participants will allow these data on the distribution of developmental disorders to be extended to the general Italian pediatric population. One main limitation of the study is the lack of clinical confirmation of the data yielded by the screening programme, which the authors aim to obtain in later stages of the study.

## Introduction

Developmental disorders encompass a broad spectrum of disabilities, which are characterized by difficulties with language and speech, motor skills, behavior, memory, learning, and other neurological functions. These disorders are likely to result from a combination of genetic, biological, psychosocial, and environmental risk factors (1).

Early identification of developmental disorders is crucial to children well-being and to provide appropriate therapies and education (2).

Recent studies suggest periodic developmental surveillance, screening, evaluation, and re-evaluation throughout childhood (3, 4) including the Parental Evaluation of Developmental Status (5). Nevertheless, the most effective way to identify children with developmental disorders remains elusive (6, 7).

The American Academy of Pediatrics (8) recommends the screenings at different age stages and according to different aims: during the first year of life of the child, paying particular attention to postpartum depression in mothers; at 9, 18, and 24 or 30 months of age, for the early identification of developmental disorders; at 18 and 24 months, for the early identification of autism; at 4 years, to investigate the prerequisites for access to preschool; from 5 years, to evaluate mental health and psychosocial functioning.

Several epidemiological studies have evaluated the rate of developmental disorders, though the results of such studies have proved to be somewhat varied.

In the USA, for instance, studies have reported that 12–16% of children suffer from developmental disorders (6, 9, 10). By contrast, studies conducted in other countries (such as Peru, Malaysia, the Netherlands, Norway, Korea) have reported that these problems affect 5–10% of the pediatric population (11–17). Mackrides and Ryherd (18) reported that the number of children under 5 years of age that display a developmental disorder ranges from 2 to 16%, and that approximately half of these receive a late diagnosis. Other studies have reported that between 4 and 10% of children in the general population have developmental disabilities, though only 30% of these are identified before primary school (19, 20). A recent study (21) in Denmark reported a 1/186 ratio of subjects with relative risk of autism spectrum disorders (ASDs) in the general population. No data are available on developmental disorders in the general pediatric population for Italy. Data on the prevalence of developmental disorders in the clinical population (from local mental health services for outpatients in the Emilia Romagna region) yielded percentages of 7.79% for cognitive disability, of 3.04% for ASDs, of 17.39% for language delay, and of 1.53% for developmental coordination disorder (22).

Another study carried out in the Piemonte region^1^ yielded a percentage of 4.81% for developmental disorders in the overall patient population in neuropsychiatry services. Moreover, the first Italian data that have been released on the prevalence of ASD by the Piemonte, Veneto, and Emilia Romagna regions indicate that the percentage of children with ASD ranges from 1.8 to 3.1% in a population aged from 1 to 18 years.^2^

### The “Osserviamo” Project

On the basis of these considerations, the Rome Municipality and the Department of Pediatrics and Child Neuropsychiatry at Sapienza University of Rome are coordinating the “Osserviamo” project. The project has three objectives: to validate a tool on the Italian population for the screening of developmental disorders; to collect a first set of epidemiological data on the early identification and prevention of developmental disorders in a group of 3- to 4-year-old children; and to create a network of collaboration between parents, kindergarten, and neuropsychiatry services. Data are being collected by Ages and Stages Questionnaire-3rd Edition (ASQ-3), a self-report questionnaire that had demonstrated good psychometric properties for the general pediatric population (9). The aim of the project is to collect data from 4,000 subjects over a 5-year period starting from 2012 (about 800 for each year), which means that the data collection is still in progress. We decided to focus on 3- to 4-year-old children because several conditions of delayed or mild disability may not be evident at early age and may emerge when a child is confronted with more demanding developmental challenges, such as the start of kindergarten.

### Objectives of this Paper

In this paper, we present the preliminary data from the first year of implementation of the “Osserviamo” project. Specifically the aims of the present work were:

1. To verify the appropriateness of the method adopted, and in particular of the sample recruitment method and of the self-report measure administered to parents. In other screening studies (4, 23) samples were recruited by pediatric health care professionals, whereas in the “Osserviamo” project the sample was recruited from schools. Since children in Italy usually start kindergarten at 3 years of age, the authors hypothesized that the recruitment of children in schools would allow a representative sample of the entire pediatric population in this age group to be contacted. We calculated what percentage of the sample contacted agreed to participate in the study. Moreover, parents were asked to complete a self-report questionnaire on developmental delay: the percentage of correctly completed questionnaires was also calculated.
2. To analyze a first data set resulting from the screening observation (600 subjects). As no Italian validation of the ASQ-3 is yet available, our results are based on the American validation cutoff scores of the tool. With regard to this point, the authors specifically aimed to:
  – observe the distribution of ASQ-3 scores and the percentages of children identified as “not at risk,” “at risk” and “clinical” by the ASQ-3 scores (in different domains of the ASQ-3), according to gender and age groups;
  – evaluate differences between males and females in the different domains of the ASQ, separately for the two age groups;
  – evaluate false positives through a clinical evaluation of the children whose score fell within the clinical range in at least one ASQ-3 domain.

## Materials and Methods

### Sample Selection

During the first year of implementation of the study (2012), a sample of 600 children aged from 39 to 50 months was identified in 15 kindergarten schools of Rome (one for each sub-municipality of Rome). The schools and the number of children for each school were selected according to the most recent population census, conducted in Rome in 2011, which provided information on the number of inhabitants in each of the 15 sub-municipalities of Rome and the number of children in each school in the sub-municipalities (24). A representative sample of children (proportionate to the number of children living in each sub-municipality) aged between 39 and 50 months (i.e., attending the first year of kindergarten) was randomly selected in each of these schools, thus ensuring that the sample was composed of children from all the sub-municipalities. This method does not, however, guarantee that all the socioeconomic levels were represented equally. This variable will be controlled more systematically in the subsequent stages of the Osserviamo project than was possible in this first step of the study.

### Procedure

Once we had identified the sample, we obtained authorization from the school districts to contact the schools’ principals. Three meetings with the schools’ principals and the teachers’ coordinators were held to explain what developmental disorders are, as well as the aims and the methodology of the Osserviamo project. All of the principals agreed to the project and consequently allowed us to contact the children’s parents through the teachers’ coordinators, who ensured that the questionnaire was sent to and returned by the parents. Informed consent was obtained from the parents of children who agreed to participate in the study. We requested an e-mail address or telephone number to be able to contact them again. All the parents were informed that the information collected would be confidential and that participation in the study was exclusively on a voluntary basis. A dedicated telephone number was also set up so as to answer any questions the parents wished to ask about the Osserviamo project. The parents were then asked to complete the Ages and Stages Questionnaire-3 (see Assessment). All the data were collected in the school year 2011–2012.

In a second step, the parents of children who fell within the clinical range in one, or more than one, domain of the ASQ-3 were contacted by email or telephone by a clinician of the neuropsychiatric services, who suggested that a meeting be arranged to discuss the ASQ-3 data. This first observation was followed, if deemed necessary, by a comprehensive neuropsychiatric evaluation (cognitive, neuropsychological, and psychopathological) of the child, during which the disorder was either confirmed or not confirmed. Children were thus offered the opportunity to promptly start targeted treatment that involved both the parents and schools directly.

### Assessment

The authors used the 42-month (from 39 to 45 months) and the 48-month (from 45 to 51 months) forms of the Ages and Stages Questionnaire-3rd Edition (ASQ-3).

The ASQ-3 is a screening tool used to assess development during the first 5 years of life (20). It consists of 21 questionnaires to be used on children of 2, 4, 6, 8, 9, 10, 12, 14, 16,18, 20, 22, 24, 27, 30, 33, 36, 42, 48, 54, and 60 months of age. The questionnaires have 30 developmental items divided into five domains: communication, gross motor, fine motor, problem solving, and personal–social development. The questionnaire must be completed by the parent or the caregiver of the child. Answers to items are rated *yes, sometimes*, or *not yet* and are scored as 10, 5, or 0, respectively. Each questionnaire is administered to the children of that age and in the ±1-month range. As the questionnaires cover wider age ranges after 27 months, administration of two successive forms is recommended for children who are outside the ±1-month range (20). Domain cutoff scores were calculated on the basis of 2 SD. It was suggested that children whose questionnaire results stood at, or below, the established cutoff score in one or more of the domains should be referred for further assessment.

Several reports indicate that the ASQ-3 has well-established psychometric properties in a clinical context, such as test–retest reliability, internal consistency, criterion validity, sensitivity, and specificity (20, 25–28). The validity of the ASQ-3 tested on 18,000 questionnaires administered in the United States yielded an overall agreement across questionnaires of 86%, with a range of 73–100%. Sensitivity (i.e., children in whom the ASQ detected a developmental delay and who had a delay according to the standardized assessment) ranged from 85 to 92%, while specificity (i.e., children in whom the ASQ-3 did not detect a delay and whose development was normal according to the standardized assessment) ranged from 78 to 92%. Validity and reliability ranged from 70 to 100% (25–28).

In the present study, since an Italian version of the ASQ is not available, the questionnaire was translated from English into Italian and, subsequently, to verify any language inaccuracies, it was back translated into the original language. A pilot administration to 20 parents permitted some cultural and lingual adaptations. The final version was therefore included in the study.

### Data Analysis

In order to evaluate the sample recruitment method and the use of the self-report measure administered to parents, we calculated the percentage of the sample who agreed to participate in the study and who completed the questionnaires correctly.

In order to analyze a first set of data yielded by the screening program, we calculated the distribution of the scores as well as the percentages of children identified as being “not at risk,” “at risk,” and “clinical,” according to gender and across age stages, on the basis of the USA cutoff values. A series of Student’s *t*-test were used to compare the scores of the gender groups (boys vs girls) on different ASQ-3 domains, and a Chi^2^ test was performed to verify difference between the two age stages on the prevalence of subjects with a score in the clinical range. The clinical diagnosis made in the children who fell within the clinical range of the ASQ-3, and consequently underwent a neuropsychiatric evaluation, was provided, as was the percentage of false positive of the ASQ-3.

The data were analyzed by means of the SPSS program.

## Results

Data were collected from 532 out of a total of 600 children (88.67% of the sample agreed to participate) and were analyzed in 514 children (3.38% of the questionnaires were not answered correctly). The reasons for why parents refused to participate are unknown as the authors were unable to contact them.

The whole sample was divided into two age stages: 39–45 months (242 subjects; 123 boys and 119 girls; age mean = 42.65; SD = 1.82) and 45–51 months (272 subjects; 147 boys, 125 girls; age mean = 48.08; SD = 2.62).

Table 1 shows the mean values and SD for each ASQ-3 domain for the whole sample divided according to age stages for both the Italian and USA studies, and divided according to gender for the Italian sample alone. From a qualitative point of view, the means of the Italian sample were higher than those of the USA sample, while the Italian SDs were lower than the USA SDs, in each ASQ-3 domain and for both the 42- and 48-month age stages. As regard the 42-month age stage, Student’s *t*-tests between gender groups revealed significant differences in fine motor skills (*t* = −2.91; *p* = 0.002; degrees of freedom = 240; boys mean = 52.83, SD = 9.16; girls mean = 56.26, SD = 7.60) and personal–social development (*t* = −2.41; *p* = 0.02; degrees of freedom = 240; boys mean = 50.70, SD = 8.01; girls mean = 53.28, SD = 7.85). In particular, boy scores were lower than girl scores in both domains. As for the 48-month age stage, boy scores were lower than girl scores in fine motor skills alone (*t* = −3.50; *p* = 0.002; degrees of freedom = 270; boys mean = 50.34, SD = 11.52; girls mean = 54.92, SD = 7.81).

**Table 1:** Means and SD for each Ages and Stages Questionnaire-3rd Edition (ASQ) domain, for the USA and Italian samples, separately for 42- and 48-month age stages.

	Communication	Gross motor	Fine motor	Problem solving	Personal–social development

ASQ age stage: 42 months					
**Total USA sample, *n* = 956**					
Mean	50.02	54.03	47.55	51.54	51.39
SD	11.48	8.88	13.87	11.72	10.13
**Total Italian sample, *n* = 242**					
Mean	57.00	56.82	54.42	56.49	51.92
SD	6.59	6.01	8.71	6.67	8.04
**Italian boys, *n* = 123**					
Mean	56.76	56.27	52.83	56.15	50.70
SD	5.89	6.51	9.16	5.74	8.01
**Italian girls, *n* = 119**					
Mean	57.23	57.35	56.26	56.81	53.28
SD	7.27	5.44	7.60	7.53	7.85

**ASQ age stage: 48 months**					

**Total USA sample, *n* = 672**					
Mean	52.92	52.71	45.35	52.78	50.34
SD	11.10	9.97	14.77	10.74	11.87
**Total Italian sample, *n* = 272**					
Mean	56.65	54.03	52.39	54.82	51.60
SD	6.34	8.42	10.25	7.79	8.25
**Italian boys, *n* = 147**					
Mean	56.12	54.66	50.34	54.18	50.75
SD	6.99	7.76	11.52	7.50	8.20
**Italian girls, *n* = 125**					
Mean	57.34	53.27	54.92	55.65	52.62
SD	5.43	9.15	7.81	8.06	8.25

*Note: means and SD for the USA samples were derived from Squires et al. (20)*.

Overall, 120 children (23.35% of the total sample of 514 subjects) fell within the risk range of at least one developmental area, while 40 children (7.78%) fell within the clinical range according to the ASQ. In particular, in the 42-month age stage, 58 children (23.97% of 242 subjects) fell within the risk range and 14 (5.79%) within the clinical range, whereas in the 48-month age stage, 62 children (22.79% of 272 subjects) fell within the risk range and 26 (9.55%) within the clinical range. As regard the clinical range, the difference between the two age stages on the prevalence of the disorders (5.79% in the 42-month age stage vs 9.55% in the 48-month age stage) was not statistically significant.

Tables 2 and 3 show the children’s developmental status for the 42- and 48-month age stages for the sample taken as a whole and divided according to gender group.

**Table 2:** Children’s developmental status in the 42-month age stage, for the whole sample and according to gender group.

	Developmental status		

	Not at risk, *n* (%)	At risk, *n* (%)	Clinical, *n* (%)
**Communication**			
Total sample, *N* = 242	236 (97.5)	5 (2.1)	1 (0.4)
Boys, *N* = 123	120 (97.6)	3 (2.4)	/
Girls, *N* = 119	116 (97.5)	2 (1.7)	1 (0.8)
**Gross motor**			
Total sample, *N* = 242	220 (90.9)	20 (8.3)	2 (0.8)
Boys, *N* = 123	107 (87%)	15 (12.2%)	1 (0.8)
Girls, *N* = 119	113 (95.0)	5 (4.2)	1 (0.8)
**Fine motor**			
Total sample, *N* = 242	234 (96.7)	6 (2.5)	2 (0.8)
Boys, *N* = 123	116 (94.3)	6 (4.9)	1 (0.8)
Girls, *N* = 119	118 (99.2)	/	1 (0.8)
**Problem solving**			
Total sample, *N* = 242	233 (96.3)	7 (2.9)	2 (0.8)
Boys, *N* = 123	117 (95.1)	6 (4.9)	/
Girls, *N* = 119	116 (97.5)	1 (0.8)	2 (1.7)
**Personal-social skills**			
Total sample, *N* = 242	215 (88.8)	20 (8.3)	7 (2.9)
Boys, *N* = 123	107 (87.0)	12 (9.8)	4 (3.3)
Girls, *N* = 119	108 (90.8)	8 (6.7)	3 (2.5)

**Table 3:** Children’s developmental status in the 48-month age stage, for the whole sample and according to gender group.

	Developmental status		

	Not at risk, *n* (%)	At risk, *n* (%)	Clinical, *n* (%)
**Communication**			
Total sample, *N* = 272	259 (95.2)	10 (3.7)	3 (1.1)
Boys, *N* = 147	138 (93.9)	7 (4.8)	2 (1.4)
Girls, *N* = 125	121 (96.8)	3 (2.4)	1 (0.8)
**Gross motor**			
Total sample, *N* = 272	245 (90.1)	18 (6.6)	8 (2.9)
Boys, *N* = 147	136 (92.5)	8 (5.4)	3 (2.0)
Girls, *N* = 125	110 (88.0)	10 (8.0)	5 (4.0)
**Fine motor**			
Total sample, *N* = 272	256 (94.1)	12 (4.4)	4 (1.5)
Boys, *N* = 147	134 (91.2)	10 (6.8)	3 (2.0)
Girls, *N* = 125	122 (97.6)	2 (1.6)	1 (0.8)
**Problem solving**			
Total sample, *N* = 272	253 (93.0)	11 (4.0)	8 (2.9)
Boys, *N* = 147	134 (91.2)	9 (6.1)	4 (2.7)
Girls, *N* = 125	119 (95.2)	2 (1.6)	4 (3.2)
**Personal-social skills**			
Total sample, *N* = 272	257 (94.5)	11 (4.0)	3 (1.1)
Boys, *N* = 147	137 (93.2)	8 (5.4)	2 (1.4)
Girls, *N* = 125	121 (96.8)	3 (2.4)	1 (0.8)

As regard the clinical evaluations, 9 children in the 42-month age stage (out of a total of 14 children who fell within the clinical range) and 15 children in the 48-month age stage (out of a total of 26 children who fell within the clinical range) underwent a neuropsychiatric evaluation and thus received a clinical diagnosis. Sixteen parents refused the clinical evaluation: 12 of these parents (4 from the 42-month age stage and 8 from the 48-month age stage) did not agree to the clinical evaluation because their children had already received a diagnosis and were being treated in other services, whereas the remaining 4 did not provide any reason for their refusal. As regard children who had already received a diagnosis, 3 in the 42-month age stage were diagnosed with a Language Disorder and 1 with a Language Disorder and a comorbid Developmental Coordination Disorder; 7 in the 48-month age stage were diagnosed with a Language Disorder and 1 with global developmental immaturity.

In the 42-month age stage sample, 1 child was diagnosed with ASD, 5 children with a Language Disorder, 2 children with a Developmental Coordination Disorder, while no diagnosis was made in 1 child; in the 48-month age stage sample, 1 child was diagnosed with ASD, 2 children with a Language Disorder, 4 children with a Developmental Coordination Disorder, 2 children with a Intellectual Disability, 1 child with a Developmental Coordination Disorder plus Intellectual Disability, 3 children with a Language Disorder plus Developmental Coordination Disorder, while non-diagnosis was made in 2 children.

In conclusion, 3 children (out a total of 24 children evaluated, 12.50%) who fell within the clinical range of the ASQ-3 did not receive a diagnosis (false positive); all 3 of these children displayed a range of difficulties and developmental immaturity.

## Discussion

The first objective of this study was to assess whether the methods adopted to recruit the sample of the screening program were adequate, and whether the school context provided favorable recruitment conditions for the study.

Another reason for the choice is that the recruitment of children through schools helps to create a direct relationship between parents and the school and neuropsychiatric services, thereby providing support to parents and teachers as well as direct access to these services when required. The data yielded by the present study on this aspect of the program confirm not only that the school system responded actively to the project (no school principal refused to cooperate), but also that a large proportion of the parents participated in the study, completing the questionnaire and returning it to the school: 88.67% (532 out of a total of 600) adhered to the screening program. This figure is in keeping with those reported by other screening programs in the literature (29). In this regard, since participation in screening programs is voluntary, a certain amount of non-adherence is to be expected (30).

A second issue that the authors investigated regarding the screening methodology was the feasibility of distributing a questionnaire to be completed by parents at home, with no direct support from a specialist. In this preliminary phase of the study, only 3.38% of the questionnaires (18 out of 532) could not be used because they had been completed incorrectly. The authors may therefore conclude that the collection of information by asking participants to complete the ASQ-3, according to instructions given to parents in writing by teachers, was effective. In addition, the low proportion of incorrectly completed questionnaires indirectly confirms that the questionnaire was relatively easy to complete and that it allowed, by investigating the children’s skills in daily life, parents to conduct a guided observation of their children’s behavior and of their development stage. This tool can thus be easily used by both families and teachers.

Another objective of the pilot project was to analyze the preliminary screening data. Before addressing this topic, it is necessary to once again point out that no normative data for the ASQ-3 exist for the Italian population (obtaining such data is one of the general objectives of the “Osserviamo” project). This means that these preliminary data are based on guidelines drawn up according to the validation of the tool in the USA.

One aspect that deserves consideration is that regarding the averages and the SD of the US and Italian samples. A substantial overlap emerged between the average values in the age groups considered, although the values in the Italian sample are always higher than those in the US sample. The Italian values are actually closer to those yielded by other validations of the ASQ-3 [for example, in Korean and Norway (17)] than to the American values. As regard the SD, however, the Italian values appear to be qualitatively lower than those of the US. This becomes even more significant since the cutoff for being defined either “at risk” or “clinical” is based on the rule of the mean minus 1 or 2 SD; the cutoff used to define the risk and clinical areas in the selected sample is consequently strongly dependent on these values. According to the formula used to define the cutoff, if a lower SD were to persist even when the size of the Italian sample increases, the authors could assume that the Italian cut-off values need to be lower than the US cutoff values. This would alter the results regarding the frequency of “at risk” and “clinical” children in the study population in this preliminary phase of the study (in the 42-month range, 24% of the sample falls within the risk range, and about 6% in the clinical range, whereas in the of 48-month range, about 23% of the sample falls within the risk range, and about 10% in the clinical range) since the adoption of the US cutoff may have led to the phenomenon being underestimated in the Italian population.

Moreover, with regard to gender differences, which are obtained by comparing the average boy and girl scores and are therefore not likely to be affected by the use of the US cutoff, girl scores were significantly higher than those of boys (with the former consequently appearing to be more competent) in fine motor skills and in personal–social skills in the 42-month range as well as in fine motor skills in the 48-month range. These data are in keeping with those reported in other studies based on the ASQ-3 (e.g., Brazilian), in which girl subjects achieved higher scores than boys (31, 32). These results also reflect the findings which indicate that girls from different cultural backgrounds in the preschool age group achieve higher scores in all areas except gross motor skills (30). The fact that the results yielded by this preliminary screening program are in keeping with those of other study points to the validity of the adopted tool (30–35).

As regard the clinical evaluations, the parents of 24 (out of a total of 40) of the children who fell within the clinical range at the ASQ-3 agreed to the clinical evaluation. Some of the parents who refused did not agree the clinical evaluation because their children had already received a diagnosis and were being treated in other services, whereas others did not provide any reason for their refusal. The fact that the most frequent disorder in children in whom a diagnosis has already been made is the language disorder (10 children out of a total of 12) is likely to be due language difficulties being more easily recognized than other difficulties by adults, who then seek help. Some of these children scored in the clinical range in other ASQ-3 domains, such as in the personal–social skill; it is possible that children being treated for language difficulties are perceived by their parents as being less skillful on the social level than on the language level because the children’s language level is currently closer to normal, or perceived as being closer to normal, than their social level as a result of the therapy.

The children who were evaluated and received a neurodevelopmental diagnosis accounted for 87.50% of the sample (12.50% of false positives). Preliminary data on specificity of the ASQ-3 indicate that the trend is good, though no statistical analysis can be performed yet owing to the small sample size. As regard the clinical diagnosis, 2 children were diagnosed with ASD; the identification of these two children between 42 and 48 months of age with undiagnosed autism and the prompt activation of support for the children, families, and teachers is highly relevant to the children’s future development. Moreover, language disorders were predominant in the 42-month age stage (5 children out of a total of 9), whereas they decreased (2 children out of a total of 15) in the 48-month age stage or occurred in comorbidity with other disorders, such as the developmental coordination disorder. Overall, language difficulties appear to be more visible to a child’s parents, whereas coordination disturbances escape recognition more often and are consequently diagnosed less often as a result of parental reports.

One of the main limitations of this study was the use of normative data taken from the US validation of the questionnaire. Indeed, our data were analyzed using the US cutoff values, which were drawn up for a very different population in terms of composition, sociodemographic characteristics and cultural activities. The potential bias due to the use of this tool may be even greater since the etiology of developmental disorder is multifactorial and depends on environmental and genetic factors that impact in different ways on a child’s phenotype. Although the authors were obliged to use this cutoff value in the preliminary phase of the data analysis, calibration of the tool is one of the objectives of the “Osserviamo” project.

Other limitations of the present study are the lack of the clinical confirmation of the data yielded by the screening program, which the authors aim to obtain in later stages of the study, and the fact that the children sample was limited to Rome, which is the only municipality in which funding for data collection was available.

In the future, the “Osserviamo” project, which is an ongoing study, aims to study a larger sample (*N* = 4,000), to validate the ASQ-3 and to perform a clinical study on children defined as being either “at risk” or “clinical” by the screening program.

## Ethics Statement

The design of the screening was approved by the ethical committee of Department of Dynamic and Clinical Psychology, “Sapienza” University of Rome, Italy.

## Author Contributions

CS had primary responsibility for protocol development and patient enrollment, supervised the execution of all the phases of the screening, and contributed to the writing of the manuscript. EC participated in the development of the protocol and had primary responsibility for patient screening; MT analyzed the data. FG, FM, LN, FP, VS, and LV contributed equally to the screening and interpretation of data. All authors contribute to the writing of the manuscript. All authors read and approved the final version of the manuscript.

## Conflict of Interest Statement

The authors declare that the research was conducted in the absence of any commercial or financial relationships that could be construed as a potential conflict of interest.

## Footnotes

^1^www.sinpia.eu/atom/allegato/1052.pdf.

^2^www.epicentro.iss.it.
